# Real-World Data on the Efficacy and Safety of Osilodrostat in Patients with Cushing’s Disease in Spain

**DOI:** 10.3390/jcm14217575

**Published:** 2025-10-25

**Authors:** Marta Araujo-Castro, Rogelio García-Centeno, Laura González, Felicia A. Hanzu, Aida Orois, Rosa Camara, María Dolores Ollero García, Ana Irigaray Echarri, Paola Gracia Gimeno, Eider Pascual-Corrales, Betina Biagetti, Andrés Cardona, Inmaculada González Molero, Andreu Simo-Servat, Fernando Guerrero Pérez, Rocío Villar-Taibo, Ignacio Bernabéu, Carmen Fajardo-Montañana, Cristina Novo-Rodríguez, Carmen Tenorio Jimenéz, María Calatayud, María Dolores Moure Rodríguez, Fernando Cordido, Ana Castro, Lucía Manzano Valero, Miguel Paja Fano, Jessica Goi, Anna Aulinas, Pablo Abellán, Pedro Iglesias, Alfonso Soto-Moreno

**Affiliations:** 1 Endocrinology & Nutrition Department, Hospital Universitario Ramón y Cajal & Instituto de Investigación Biomédica Ramón y Cajal (IRYCIS), Colmenar Viejo Street km 9, 28034 Madrid, Spain; 2 Endocrinology & Nutrition Department, Hospital Universitario Gregorio Marañón, 28007 Madrid, Spain; 3 Endocrinology & Nutrition Department, Hospital Clinic de Barcelona, 08036 Barcelona, Spain; 4 Endocrinology & Nutrition Department, Hospital La Fe, 46600 Valencia, Spain; 5 Endocrinology & Nutrition Department, Hospital Universitario Navarra, 31008 Pamplona, Spain; 6 Endocrinology & Nutrition Department, Hospital Universitario Royo Villanova, 50015 Zaragoza, Spain; 7 Endocrinology & Nutrition Department, Hospital Universitario Vall de Hebrón & CIBERER Group 747, 08035 Barcelona, Spain; 8 Endocrinology & Nutrition Department, Hospital Regional Universitario de Málaga, 29010 Málaga, Spain; 9 Endocrinology & Nutrition Department, Hospital Regional Universitario de Terrasa, 08227 Barcelona, Spain; 10 Endocrinology & Nutrition Department, Hospital Universitario de Bellvitge, L’Hospitalet de Llobregat, 08907 Cataluña, Spain; 11 Endocrinology & Nutrition Department, Hospital Universitario de Santiago de Compostela, 15706 Galicia, Spain; 12 Endocrinology & Nutrition Department, Hospital Universitario La Ribera, 46600 Valencia, Spain; 13 Endocrinology & Nutrition Department, Hospital Universitario Virgen de las Nieves, 18014 Granada, Spain; 14 Endocrinology & Nutrition Department, Hospital Universitario 12 de Octubre, 28041 Madrid, Spain; 15 Endocrinology & Nutrition Department, Hospital Universitario de Cruces, 48903 Bilbao, Spain; 16 Endocrinology & Nutrition Department, Hospital Universitario A Coruña & Universidad de A Coruña, 15006 A Coruña, Spain; 17 Endocrinology & Nutrition Department, Hospital Universitario Toledo, 45007 Toledo, Spain; 18 Endocrinology & Nutrition Department, OSI Bilbao-Basurto, Hospital Universitario de Basurto, 48013 Bilbao, Spain; 19 Medicine Department, University of the Basque Country UPV/EHU, 48013 Bilbao, Spain; 20 Endocrinology & Nutrition Department, Hospital de la Santa Creu i Sant Pau, IR-SANT PAU, CIBERER U747 (ISCIII), 08025 Barcelona, Spain; 21 Endocrinology & Nutrition Department, Hospital Universitario de Valencia, 46010 Valencia, Spain; 22 Endocrinology & Nutrition Department, Hospital Universitario Puerta de Hierro, 28222 Madrid, Spain; 23 Unidad de Gestión de Endocrinología y Nutrición, Instituto de Biomedicina de Sevilla (IBiS), Hospital Universitario Virgen del Rocío/CSIC/Universidad de Sevilla, 14004 Córdoba, Spain

**Keywords:** Cushing’s syndrome, Cushing’s disease, pituitary, osilodrostat, urinary free cortisol

## Abstract

**Objective**: To evaluate the efficacy and safety of osilodrostat in patients with Cushing’s disease (CD). **Methods:** A retrospective, multicenter, real-world study of patients with CD. The main efficacy endpoint was the proportion of patients who were complete responders (urinary free cortisol [UFC] < the upper limit of normal and/or with adrenal insufficiency development). **Results:** Thirty-seven CD patients were enrolled. There were 33 patients who initially received osilodrostat in monotherapy and 4 in combination. However, 3 patients of the monotherapy group were switched to combination therapy. The median duration of osilodrostat treatment was 5 months (range 1–93). All the patients were classified as responders: 33 (89.2%) had complete response and 4 partial response. A positive correlation was detected between the percentage of UFC decrease and the maximum (r = 0.481, *p* = 0.006) and the maintenance doses (r = 0.440, *p* = 0.011). The initial doses of osilodrostat were a predictor of complete response (vs. partial) (Odds ratio [OR] 2.82, *p* = 0.030). The median time to UFC normalization in the group of complete responders was 4 weeks (range 1–20) and UFC normalized before or at month 1 in 67% (*n* = 20/30) of the patients. Osilodrostat led to a significant decrease in systolic and diastolic blood pressure in parallel with a reduction of antihypertensive medications. **Conclusions:** Osilodrostat leads to a complete UFC normalization in up to 90% of the patients with CD, in parallel with an improvement in the cardiometabolic profile. A proper titration of osilodrostat is important to achieve a complete response since a positive correlation between the doses and the UFC reduction was observed.

## 1. Introduction

Endogenous hypercortisolism is a rare chronic and systemic clinical condition caused by long-term and inappropriate exposure to glucocorticoids [1]. The three main causes of endogenous Cushing’s syndrome (CS) are Cushing’s disease (CD) in 65% of the cases, adrenal CS in 30%, and ectopic CS in 5% [2].

Pituitary surgery is considered the first-line treatment for CD; however, a substantial proportion of patients may still require medical treatment [3]. The main goals of medical therapy are to normalize cortisol secretion, minimize clinical symptoms and comorbidities, improve quality of life, and reduce mortality. Ketoconazole, levoketoconazole, metyrapone, osilodrostat, mitotane and etomidate are the available adrenal steroidogenesis inhibitors for the treatment of CS in clinical practice [3,4]. Etomidate is the only available drug for parenteral administration. The reported efficacy, defined as urinary free cortisol (UFC) normalization, is around 70% for ketoconazole [5], 50% for levoketoconazole [6], 50% for metyrapone [7], and 80% for osilodrostat [8]. On the other hand, the rate of UFC normalization with pituitary-acting drugs, cabergoline and pasireotide, is low, at approximately 35% [9,10] and 45% [10], respectively. Additionally, osilodrostat and etomidate are the drugs with the fastest onset of action, leading to rapid control of hypercortisolism. Thus, to date, the most effective oral drug for controlling hypercortisolism is osilodrostat. However, osilodrostat remains an underused treatment in Spain, mainly due to restrictions on its use.

Osilodrostat is an imidazole derivate that inhibits 11β-hydroxylase (CYP11B1) and aldosterone synthase (CYP11B2) [11]. Several phase II and III clinical trials, the LINC trials, have proved its high efficacy in patients with CD, showing reductions in UFC along with significant improvements in body weight, blood pressure (BP), glucose metabolism, lipid profile, psychological status and quality of life [8,12,13,14]. However, few data exist about the real-world efficacy of osilodrostat in patients with CD [15,16], and most of the data is limited to case reports [17,18]. Considering this background, we aimed to evaluate the efficacy and safety of osilodrostat, both in monotherapy and in combination with other treatments, for the treatment of patients with CD in Spain.

## 2. Materials and Methods

### 2.1. Study Design and Definitions

**Figure 1:**
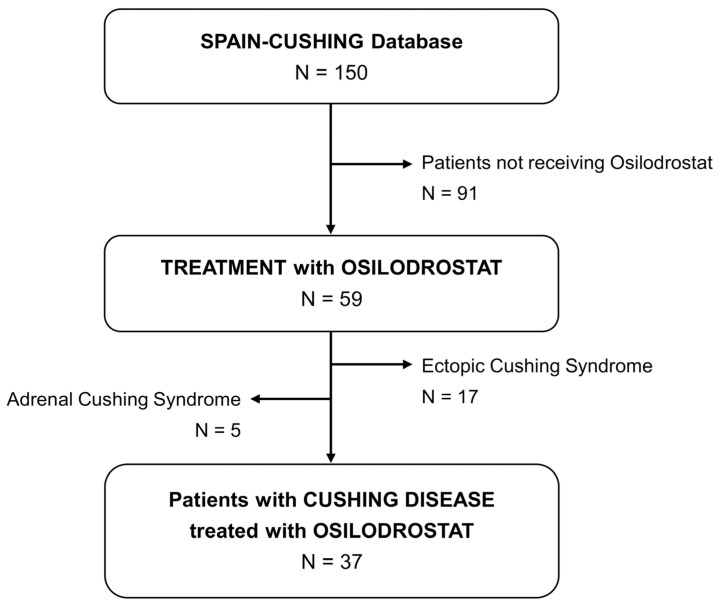
Study population.

A multicenter, retrospective study was designed to evaluate patients with CD treated with osilodrostat. Members of the Neuroendocrinology Area of the Spanish Society of Endocrinology and Nutrition (SEEN) were invited to participate and contribute patient data for inclusion in the SPAIN-CUSHING database, which compiles real-world evidence on patients with endogenous CS across multiple centers in Spain. A sub-analysis of this national database was conducted to focus specifically on patients with CD receiving osilodrostat. At the time of data analysis (June 2025), the database included 150 patients with endogenous CS, of whom 37 had a confirmed diagnosis of CD and had been treated with osilodrostat (Figure 1).

After confirming ACTH dependent endogenous hypercortisolism, CD diagnosis was based on the following criteria (one or more): (i) Bilateral inferior petrosal sinus sampling (BIPSS) with a petrosal sinus to peripheral ACTH ratio greater than 2:1 without CRH stimulation or higher than 3:1 after CRH stimulation from either petrosal sinus (*n* = 10), (ii) ACTH dependent CS and pituitary tumor ≥ 6 mm (*n* = 16), (iii) positive immunostaining for ACTH in the pituitary tumor (*n* = 24) and/or (iv) adrenal insufficiency after pituitary surgery (*n* = 12). These 12 cases with adrenal insufficiency developed recurrence during follow-up; and for this reason, were treated with medical therapy later.

As the study employed secondary data collection of anonymized patient data, a waiver of consent was granted by Ethical Committee of the Ramón y Cajal Hospital for those patients who do not continue follow-up in the center.

### 2.2. Primary and Secondary Outcomes

The main outcome of the study was the proportion of patients who were complete responders (mean UFC < upper limit of normal [ULN] and/or who developed adrenal insufficiency after osilodrostat initiation). As a secondary endpoint we analyzed the proportion of partial responders (mean UFC > ULN and with >50% reduction from baseline levels) and non-responders (mean UFC > ULN and with <50% reduction from baseline). In addition, we evaluated the impact of osilodrostat therapy on cardiometabolic comorbidities (type 2 diabetes, hypertension, obesity, hypokalemia and dyslipidemia), as well as the time required to achieve control of hypercortisolism.

In relation to safety, we included data on the development of adverse events (AEs). Glucocorticoid withdrawal syndrome (GWS) and adrenal insufficiency (AI) definitions were differentiated according to the symptoms reported by the patient and the level of morning serum cortisol. When symptoms were consistent with GWS and serum cortisol at the time of the event was greater than 10 μg/dL, the patient was classified as GWS. On the other hand, when cortisol levels were ≤10 μg/dL, the AE was classified as AI. We defined AI as prolonged when it persisted for more than 1 month after discontinuation of osilodrostat.

### 2.3. Variables and Hormonal Assays

UFC was measured by chemiluminescent immunoassay (CLIA), enzyme-linked immunosorbent assay (ELISA) and radioimmunoassay. As the normal range of UFC differed across center, we calculated the deviations above the ULN for each UFC values. Escape from response was defined as mean UFC above the ULN on at least two consecutive visits at the highest tolerated dose of osilodrostat, after previously achieving UFC normalization.

The individual duration and doses of previous treatments before osilodrostat (metyrapone, ketoconazole and others) were also described. In relation to osilodrostat treatment, we collected information on starting, maximum and maintenance doses. Information about the treatment strategy used (titration, titration followed by block and replace (B&R) or B&R from the beginning) was registered. Due to the regulatory restrictions in Spain, osilodrostat is not approved as a first line-therapy for CD, except under specific circumstances.

### 2.4. Statistical Analysis

The statistical analysis was performed with STATA 15 and GraphPad Prism 9.0. Categorical variables were expressed as percentages and (absolute values of variable) and quantitative variables as mean and standard deviation or median ± range depending on if the assumption of normality was met. The paired *t*-test or the Wilcoxon signed-rank test were used, as appropriate, for comparison of UFC, serum cortisol, clinical score, systolic BP (SBP), diastolic BP (DBP), BMI, and the biological parameters kalemia, fasting plasma glucose (FPG), and HbA1c before and during osilodrostat therapy. Univariable logistic regression analysis was used to estimate odds ratio (OR). Correlation between continuous variables was estimated with the Pearson correlation test. In all cases, a two-tailed *p* value < 0.05 was considered as statistically significant.

## 3. Results

### 3.1. Baseline Characteristics

Overall, 37 patients with CD (25 females and 12 males) were included. Baseline characteristics of the patients at the time of the CS diagnosis are described in Table 1. Prior to osilodrostat treatment, most patients had undergone one pituitary surgery, while 38% had undergone two surgical procedures. Moreover, 38% of the patients had received radiotherapy. In addition, 65% were treated with ketoconazole monotherapy and 27% with metyrapone monotherapy. One patient had previously been treated with cabergoline and another one with pasireotide, as steroidogenesis inhibitors are generally considered as first-line medical therapy in Spain due to their efficacy and local reimbursement policies. Overall, osilodrostat was started as first-line medical therapy in 5 cases, as second-line therapy in 17, as third-line in 11 and as fourth-line in 4 cases. The reasons for initiating osilodrostat in patients with complete response with ketoconazole and/or metyrapone were the development of AEs or loss of efficacy.

### 3.2. Osilodrostat Treatment: Efficacy and Safety

**Figure 2:**
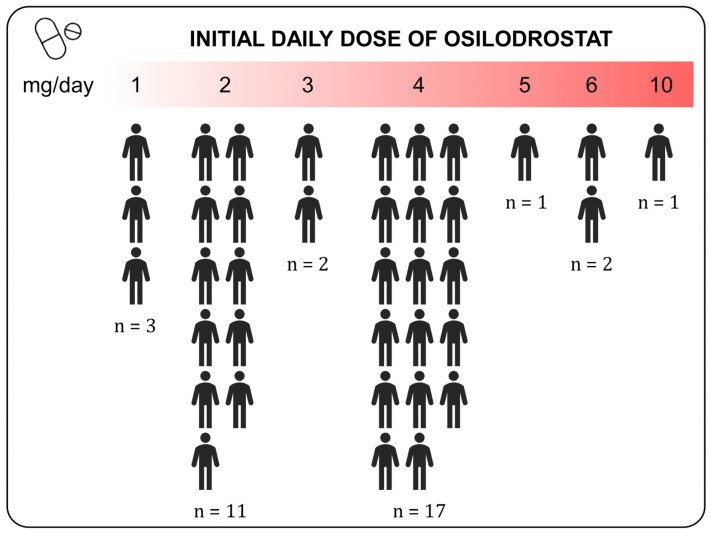
Initial daily doses of osilodrostat used in patients with Cushing disease. The “n” represents the absolute number of patients receiving the indicated daily dose of Osilodrostat.

Thirty-three patients initially received osilodrostat monotherapy, and four received combination therapy. However, three patients from the monotherapy group were later switched to combination therapy. The median initial doses of osilodrostat were 4 mg/day (range 1–10 mg/day). No differences were observed in the initial doses between the group treated in monotherapy and in combination with other drugs (*p* = 0.527). The median maximum doses were 4 mg/day (range 1–40 mg/day) and the maintenance doses 4 mg/day (range 1–30). The most common initial dose employed was 4 mg/day (Figure 2). Overall, we observed a significant increase in doses when comparing initial and maintenance doses (3.4 ± 1.72 vs. 5.4 ± 5.47, *p* = 0.020). Nevertheless, when we compared maximum initial doses and maintenance doses, the dose tended to decrease (7.4 ± 7.90 vs. 5.5 ± 5.58, *p* = 0.101). The seven cases treated with combination therapy received osilodrostat in combination with cabergoline (*n* = 6) or pasireotide (*n* = 1).

Overall, the median time from CD diagnosis to the initiation of osilodrostat was 77.4 months (range 0.5 to 274) and the median duration of the treatment with osilodrostat was 5 months (range 1 to 93). Only 3 patients were treated with B&R from the beginning (initial daily doses were 2 mg/day in two cases and 6mg/day in one), whereas the other 34 were managed with a titration approach.

All the patients experienced a UFC reduction greater than 50% or UFC normalization, and thus, were classified as responders: specifically, 33 (89.2%) had complete response and 4 displayed partial response. A complete response was achieved in all patients receiving combination therapy and in 86.7% of those receiving monotherapy.

**Figure 3:**
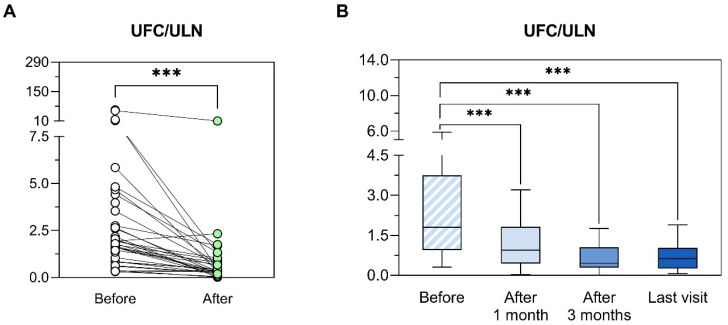
Evolution of urinary free cortisol after osilodrostat initiation. (**A**) Comparison between baseline levels of UFC/ULN and minimum UFC/ULN values observed after the initiation of the treatment with osilodrostat. For comparisons, Wilcoxon matched-pairs test was used. *** *p* < 0.001; UFC: urinary free cortisol; ULN: upper limit of normal. (**B**) Evolution of UFC/ULN before and after 1 month, 3 months and at last visit available after the initiation of the treatment with osilodrostat. UFC/ULN values were represented using Tukey box plots with 25th, 50th and 75th percentiles; whiskers extend to the most extreme data points within 1.5-times the interquartile range. The Wilcoxon matched-pairs test was applied to compare paired data. *** *p* < 0.001; UFC: urinary free cortisol; ULN: upper limit of normal.

The overall median reduction of UFC was 62% and there was a significant reduction of UFC with osilodrostat (4.5 ± 10.52 ×ULN to 0.97 ± 1.87 ×ULN, *p* = 0.034). The median time to UFC normalization in the group of complete responders was 4 weeks (range 1–20) and 67% (*n* = 20/30) of the patients had normalized UFC before or at month 1 (in three patient UFC was not measured at week 2 and 1 months, thus the time to achieve control is not available; Figure 3). No differences in the median maximum doses were found between complete and partial responders (4 mg/day [range 1–40 ] vs. 4 mg/day [range 3–15], *p* = 0.997). However, the median initial doses tended to be higher in the first group (4 mg/day [1,2,3,4,5,6,7,8,9,10] vs. 2 mg/day [1,2,3], *p* = 0.081). In fact, the initial dose of osilodrostat was a predictor of complete response (vs. partial) to osilodrostat (OR 2.82, *p* = 0.030). Pretreatment UFC levels did not predict the response to osilodrostat (OR 0.96, *p* = 0.145). A positive correlation was detected between the percentage decrease in UFC and both the maximum dose employed (r = 0.481, *p* = 0.006) and the maintenance dose (r = 0.440, *p* = 0.011).

In relation to AEs, 10 patients reported one or more AEs: 3 patients experienced hypocortisolism, 1 hyperandrogenism, 2 edemas, 1 alteration of the liver function, 3 gastrointestinal discomfort and 4 GWS (Table 2). No other AEs were reported. Six patients discontinued osilodrostat: 5 underwent surgery and achieved remission, while one discontinued therapy due to prolonged AI. Overall, prolonged AI occurred in 3 patients, one of whom had previously received radiotherapy (Table 3). The doses of osilodrostat varied among patients who developed AI, suggesting that dose did not appear to be a predictor of AI development.

Pituitary tumor remained stable after osilodrostat initiation in all patients except for a patient with a pituitary tumor with an initial microadenoma (operated in January 2003, pathological results: ki 67 of 10%, positive p53) who experience a recurrence in June 2019 with no initial visible tumor. However, a macroadenoma of 17 mm was evident 12 months after the initiation of osilodrostat. For this reason, a second surgery was performed in February 2024, with no cure. Then, the patient was submitted to radiotherapy in January 2025. Pasireotide and cabergoline were also initiated, with no proper control. Thus, in April 2025, metyrapone was added and the patient achieved UFC normalization.

### 3.3. Osilodrostat Treatment: Impact on the Control of Cardiometabolic Comorbidities

**Figure 4:**
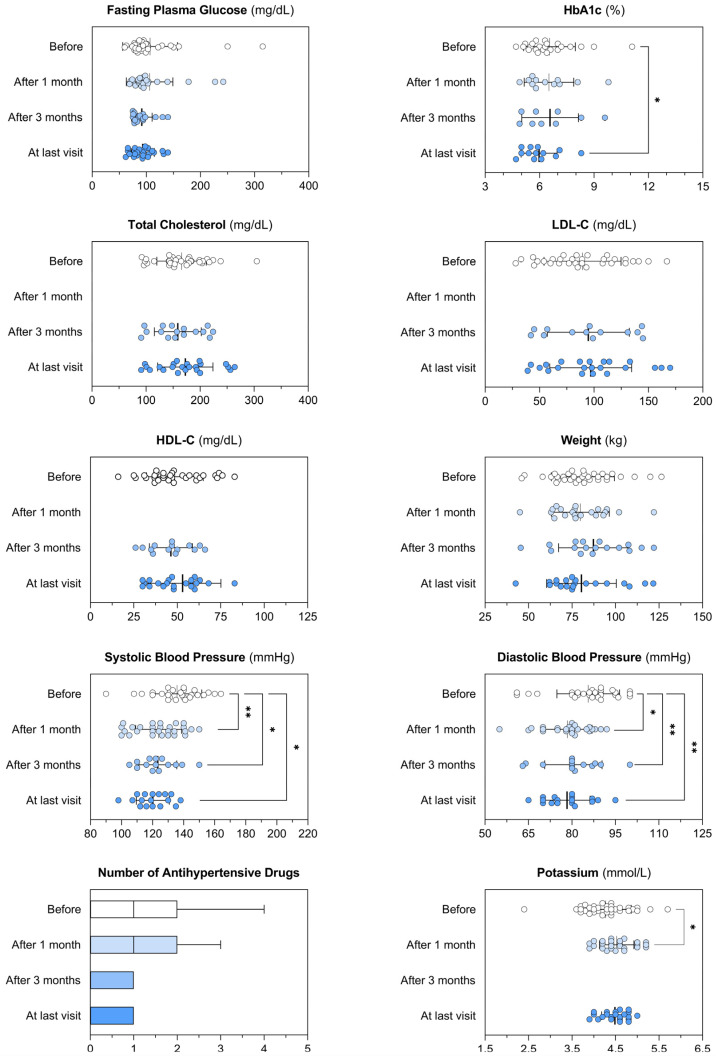
Impact of osilodrostat treatment on the control of the cardiometabolic comorbidities.

Before the initiation of osilodrostat, 35% of the patients had type 2 diabetes, 46.0% obesity, 59.5% hypertension, 28% osteoporosis and only one patient presented hypokalemia. Significant improvement in comorbidities was observed after initiation of osilodrostat, with reductions in both SBP and DBP, along with a decrease in the number of antihypertensive medications. In fact, all patients with SBP/DBP > 140/90 prior to osilodrostat treatment achieved BP < 140/90 at the last visit. In addition, there was a tendency to a reduction of HbA1c and FPG levels (Table 4 and Figure 4).

Patients’ cardiometabolic profile before and after the treatment with osilodrostat, considering different timepoints: 1 month and 3 months after the initiation of the treatment, and at last available visit. Each graph illustrates individual patient data, along with the mean and variability expressed as the standard deviation (whiskers). The number of antihypertensive drugs is shown using Tukey box plots to highlight median, interquartile range (IQR), and whiskers extending to 1.5 × IQR. Paired *t*-tests were used to compare normally distributed paired data, while the Wilcoxon matched-pairs test was applied for variables that did not follow a normal distribution. *p*-values lower than 0.05 were considered significant and illustrated within the graphs as appropriate. * *p* < 0.05; ** *p* < 0.01. HbA1c: glycated hemoglobin A1c; LDL-C: low-density lipoprotein cholesterol; HDL-C: high-density lipoprotein cholesterol.

## 4. Discussion

This is the largest real-world study focused on evaluating the efficacy and safety of osilodrostat in patients with CD. In addition, it is the first Spanish study that includes patients with CD treated with osilodrostat. Our findings are consistent with the data reported in prior clinical trials involving patients with CD (LINC trials) [8,13,14] and in other observational studies with other different populations of patients with CD, such as from EEUU [15] and Poland [16]. Our results are important since it is known that real-world observational studies provide complementary and helpful information since the drug is used based on physician criteria. In addition, the response to the treatments may be different according to the studied population. For example, previous studies have reported that Asian patients with CD required lower doses of osilodrostat than non-Asian patients to achieve therapeutic effects and developed hypocortisolism more frequently [19]. It should be noted that in our study, osilodrostat was most commonly initiated as a second- or third-line therapy, with only 5 patients receiving it as first-line medical treatment. This reflects the Spanish reimbursement criteria, as osilodrostat is approved for the treatment of endogenous CS in adults but its use is restricted to cases in which other commonly used pharmacological options, such as ketoconazole or metyrapone, are contraindicated of have failed.

In our cohort, all patients achieved at least a 50% reduction in UFC, and 89.2% reached complete biochemical normalization. Median UFC decreased by 62%, supporting and expanding on the findings of the ILLUSTRATE study [15], a retrospective real-world study conducted in the United States that included 42 patients treated with osilodrostat, 34 of whom had CD. Compared with that study, our cohort showed a slightly higher rate of biochemical normalization (89.2% vs. 70%), despite similar initial doses and titration strategies [15]. Likewise, a Polish multicenter series including 6 patients with CD treated with osilodrostat reported full cortisol normalization in 100% of patients; however, only 66.7% achieved normalization of both UFC and late-night salivary cortisol [16]. Although efficacy rates appear comparable across cohorts, the lack of precise time-to-response data in these studies limits direct comparisons. Our findings suggest that individualized titration—even from lower starting doses—can lead to excellent outcomes, particularly when guided by close and frequent biochemical monitoring without prolonging exposure to hypercortisolism. The dose–response relationship in our study revealed important patterns. Although the median values for both maximum and maintenance doses were 4 mg/day, a statistically significant increase was observed between initial and maintenance doses. The wide range of doses used across the cohort suggests considerable interindividual variability. A typical pattern emerged: initial doses were lower than both the maximum and maintenance doses, and in many cases, the maintenance dose was subsequently reduced from the peak dose achieved during titration. This trend—where the median remains stable while ranges differ—indicates the potential utility of more aggressive titration in selected patients. Moreover, the presence of a weak yet consistent correlation between UFC reduction and both maximum and maintenance doses reinforces the importance of personalized therapeutic strategies aimed at achieving normalization in cortisol levels promptly and safely. All patients receiving combination therapy achieved full biochemical control (100% vs. 86.7%), suggesting a potential synergistic effect that merits further exploration. Sixteen patients initiated osilodrostat at doses below 2 mg BID. While no significant delay in UFC normalization was observed in this subgroup, these results might suggest that in selected patient profiles—particularly those with milder hypercortisolism, older age, or multiple comorbidities—starting at a lower dose with rapid titration may be both effective and well tolerated. However, we cannot confirm this statement due to the limited number of patients in the present study prevent multiple subgroups analysis. Prospective studies are warranted to determine whether such individualized strategies optimize the balance between efficacy and safety.

Notably, the median time to UFC normalization among full responders was just 4 weeks, with 67% of patients achieving biochemical control within or before the first month. In three patients, UFC normalization occurred within the first week of treatment, underscoring the rapid onset of action of osilodrostat. This swift response is particularly relevant in clinical scenarios involving markedly elevated cortisol levels and severe hypercortisolism, where early control is crucial to prevent serious complications such as cardiovascular events, infections, and neuropsychiatric deterioration [20]. Comparative insights with other adrenal steroidogenesis inhibitors are instructive. The PROMPT study—a prospective multicenter trial evaluating metyrapone—showed that most patients achieved UFC normalization or significant reduction by week 12 [21]. However, the time to biochemical control was longer than in our cohort, and side effects such as androgenic manifestations and hypokalemia were frequently observed. Similarly, the FReSKO study, a large retrospective analysis from France involving 200 patients treated with ketoconazole, reported UFC normalization in 50% of cases and ≥50% reduction in an additional 26% [22]. Although long-term efficacy was sustained in many patients, hepatotoxicity, AI, and treatment discontinuation due to AEs represented notable limitations. The rapid biochemical control observed in our cohort—including normalization within just 7 days in certain cases—emerges as a distinctive advantage of osilodrostat. In routine clinical practice, achieving early control of cortisol excess is particularly critical in patients with severe disease, where prolonged hypercortisolism is associated with elevated risks of thromboembolic events, psychosis, and opportunistic infections. This pharmacodynamic profile supports the dual role of osilodrostat, not only as a maintenance therapy but also as a front-line option in acute settings requiring immediate cortisol reduction.

Side effects in our series were few and primarily related to AI. Two cases with long-term treatment were noteworthy, in which osilodrostat could be withdrawn without prior radiotherapy, and who persisted in remission despite more than two years without treatment. This potential long-term blocking effect of the drug during long-term use should be investigated in future studies. No specific risk factors have been clearly identified as predictors for the development of AI during osilodrostat treatment, neither the dose nor the degree of hypercortisolism. Thus, no clear explanation exists for why AI occurs in these patients; however, it is possible that individual susceptibility to osilodrostat-induced adrenocortical blockade exists in some patients with CD. No tumor growth was observed in our series, despite the complete blockade of the adrenal axis achieved in many cases. The only case of growth appears to be more related to the aggressiveness of the tumor, as observed in the histopathological study. These results are in line with those described in the LINC clinical trials [8,12,13,14].

Osilodrostat treatment led to better BP control, fewer antihypertensive medications requirement, and a trend toward lower HbA1c levels. In accordance with our results, a pooled analysis of LINC 3 and LINC 4 studies, with 210 patients with CD treated with osilodrostat included, significant reductions in mean SBP/DBP were observed from week 12 to week 72 and antihypertensive medication dose was reduced/stopped in 26.8% of patients, and the proportion taking antihypertensive medication decreased from 54.3% at baseline to 47.3% at week 72. Moreover, the study found a significant improvement in fasting plasma glucose and HbA1c in 33.3% and 61.5% at week 72, respectively [23]. Nevertheless, it should be taken into account that osilodrostat may increase mineralocorticoid precursors and lead to a deterioration of BP control [13], but this is an uncommon AE. For example, in our series of 37 patients, no cases were observed. On the pooled analysis of LINC3 and LINC4, 21.4% (*n* = 18/84) of patients with SBP ≤ 130 mmHg at baseline had SBP > 130 mmHg at week 12, and 7.2% (*n* = 9/125) of patients with DBP ≤ 90 mmHg at baseline had DBP > 90 mmHg at week 12. In our study, the reduction of SBP and DBP was evident in the first month of treatment, supporting the influence of UFC reduction in BP control.

We are aware that our study has some limitations. The main one is the short duration of follow-up after osilodrostat initiation. Additionally, due to the retrospective nature of the study, there were some missing information for clinical and biochemical parameters that may have affected the potency of some analysis. However, despite these limitations, our study represents the largest real-world investigation to date evaluating the efficacy of osilodrostat in patients with CD.

## 5. Conclusions

Osilodrostat leads to a complete normalization of UFC in up to 90% of the patients with CD, in parallel with an improvement in the cardiometabolic profile. Appropriate titration of osilodrostat is important to achieve a complete response, as there is a positive correlation between doses and UFC reduction.

## Figures and Tables

**Table 1:** Baseline characteristics at diagnosis of Cushing’s syndrome and previous treatment to osilodrostat.

Variable	Value
**Clinical and Biochemical Data**	
**Age (years)**	44.2 (range 9–75)
**Age when osilodrostat was initiated**	50.6 (range 22–79)
**Female sex**	67.6% (*n* = 25)
**Type 2 diabetes**	35% (*n* = 13)
**Hypertension**	59.5% (*n* = 22)
**Hypokalemia**	2.7% (*n* = 1)
**Fasting plasma glucose levels (mg/dL)**	103 (range 64–250)
**Serum potassium levels (mEq/mL)**	4.3 (range 2.4–5.2)
**Cortisol after dexamethasone suppression test (µg/dL)**	18.1 (3.1–52)
**UFC (×ULN)**	3.5 (range 1.1–66)
**ACTH (pg/mL)**	75.7 (range 20–134)
**Late night salivary cortisol (×ULN)**	3.1 (range 1.1–22.9)
**Previous Treatments to Osilodrostat**	
**Pituitary surgery**	81.1% (*n* = 30)
**Repeated pituitary surgery**	37.8% (*n* = 14)
**Radiotherapy**	37.8% (*n* = 14)
**Ketoconazole monotherapy** - **Median maximum doses (mg/day)** - Type of response (*n* = 23) * **Complete (UFC normalization)** **Partial (UFC reduction > 50% with high UFC)** **No response**	64.9% (*n* = 24) 600 (range 400–1600) 35% (*n* = 8) 48% (*n* = 11) 18% (*n* = 4)
**Metyrapone monotherapy** - **Median maximum doses (mg/day)** - **Type of response** **Complete (UFC normalization)** **Partial (UFC reduction > 50% with high UFC)** **No response**	27% (*n* = 10) 1000 (range 750–4000) 10% (*n* = 1) 60% (*n* = 6) 30% (*n* = 3)
**Combination therapy ^†^**	32.4% (*n* = 12)

* In one patient treated with ketoconazole, response was not evaluated since the patient developed liver function alteration, and the treatment was withdrawn. ^†^ Combination therapy included ketoconazole + metyrapone in 2, ketoconazole + cabergoline in 7, metyrapone + cabergoline in 1 and other combinations in 2 cases. ULN: upper limit of normal; UFC: urinary free cortisol.

**Table 2:** Adverse events observed during osilodrostat treatment.

Adverse Event	Frequency	Severity of the Adverse Event
**Cortisol withdrawn syndrome**	4 (10.8%)	Mild
**Hypocortisolism**	3 (8.1%)	Mild
**Gastrointestinal symptoms**	3 (8.1%)	Mild
**Edemas**	2 (5.4%)	Mild
**Hyperandrogenism**	1 (2.7%)	Mild
**Mid hypertransaminasemia**	1 (2.7%)	Mild

**Table 3:** Description of the patients who developed prolonged adrenal hypocortisolism after osilodrostat discontinuation.

Patient	Age	Sex	UFC × ULN Before Osilodrostat	Initial Daily Doses (mg)	Maximum Daily Doses (mg)	Time from Osilodrostat Initiation to AI Development	Time of AI Since Osilodrostat Discontinuation
**1**	45	F	5.9	1.0	10	57.0 months	9 months (then recovery)
**2**	60	F	2.7	2.0	2.0	75.0 months	10 months (then recovery)
**3**	22	F	2.1	4.0	4.0	20 days	7 months (then recovery)

AI: adrenal insufficiency; F: female; UFC: urinary free cortisol; ULN: upper limit of normality.

**Table 4:** Impact of osilodrostat treatment on cardiometabolic profile.

Variable	Before Osilodrostat	After Osilodrostat	Paired Data (N)	*p*-Value
**FPG (mg/dL)**	106.9 ± 50.51 (*n* = 34)	92.0 ± 30.04 (*n* = 35)	34	0.080
**HbA1c (%)**	6.5 ± 1.43 (*n* = 23)	6.1 ± 0.92 (*n* = 17)	16	0.061
**Total cholesterol (mg/dL)**	166 ± 45.8 (*n* = 34)	164 ± 47.6 (*n* = 23)	23	0.233
**LDL (mg/dL)**	90 ± 35.4 (*n* = 31)	98 ± 33.8 (*n* = 22)	21	0.242
**HDL (mg/dL)**	49 ± 16.3 (*n* = 32)	48 ± 14.5 (*n* = 22)	21	0.695
**Weight (kg)**	81.2 ± 18.13 (*n* = 33)	80.6 ± 17.63 (*n* = 33)	30	0.625
**SBP (mmHg)**	136 ± 16.0 (*n* = 28)	119 ± 12.0 (*n* = 32)	27	**<0.001**
**DBP (mmHg)**	86 ± 10.8 (*n* = 28)	75 ± 8.4 (*n* = 30)	26	**<0.001**
**Antihypertensive drugs (*n*)**	1 [0–2] (*n* = 34)	0 [0–1] (*n* = 33)	33	**0.016**
**Potassium (mmol/L)**	4.3 ± 0.59 (*n* = 34)	4.4 ± 0.36 (*n* = 32)	31	0.146

Comparison of cardiometabolic parameters before and after the initiation of osilodrostat, considering the minimum values achieved during the follow-up period. The number of patients with paired data is reported [Paired data (N)]. Paired data of normally-distributed variables were compared with Paired *t*-test. The Wilcoxon Signed-Rank test was used for non-normally-distributed variables. FPG: fasting plasma glucose; HbA1c: glycated hemoglobin A1c; LDL: low-density lipoprotein: HDL: high-density lipoprotein; SBP: systolic blood pressure; DBP: diastolic blood pressure.

## Data Availability

The data that support the findings of this study can be available by contacting the corresponding author.
